# Protection effect of thymosin β4 on ethanol injury in corneal stromal keratocyte

**DOI:** 10.1186/s12886-022-02255-8

**Published:** 2022-01-21

**Authors:** Jinghua Liu, Chen Guo, Peng Hao, Peihong Wang, Linghan Li, Yuchuan Wang, Xuan Li

**Affiliations:** 1 Nankai University, Tianjin, China; 2 Nankai University Eye Hospital, 4 Gansu Road, Heping District, Tianjin, 300020 China; 3 Tianjin Key Laboratory of Retinal Functions and Diseases, Tianjin Branch of National Clinical Research Center for Ocular Disease, Tianjin Medical University Eye Hospital, Eye Institute and School of Optometry, Tianjin, China; 4 Tianjin Key Laboratory of Ophthalmology and Visual Science, Clinical College of Ophthalmology, Tianjin Medical University, Tianjin Eye Hospital, Tianjin Eye Institute, 4 Gansu Road, Heping District, Tianjin, 300020 China; 5 Shanxi Eye Hospital, Xi’an People’s Hospital, Xi’an Fourth Hospital, Shanxi Province Xi’an, China

**Keywords:** Thymosin β4, Cornea stroma, Ethanol, Oxidative stress, Apoptosis

## Abstract

**Purpose:**

To investigate the protective effects of thymosin β4 (Tβ4) on ethanol injured human corneal keratocytes (HCKs).

**Methods:**

HCKs and BALB/c mice were chosen as the study subject. Ethanol was used to treat the cells and corneal stroma of mice to build the ethanol injured model in vitro and vivo respectively. CCK-8 was used to evaluate the cell metabolic activity. DCFH-DA was used to detect the intracellular reactive oxygen species level. TUNEL was chose to detect the cell apoptosis rate. The cell proliferation and migration were investigated by using wound healing insert. Wound healing of corneal surface and stroma was observed by using fluorescein sodium eyedrop and HE stain. RT-qPCR, ELISA, and immunostaining were performed to detect gene and protein expression in keratocytes or corneal stroma tissue of mice.

**Results:**

Ethanol induced oxidative stress injury and cell apoptosis on HCKs, and Tβ4 can alleviate it by up-regulating the expression of Bcl-2, catalase, and CuZnSOD, and inhibiting the expression of Caspase-3. Tβ4 promotes the proliferation of HCKs and the process of corneal wound healing. It may relevant to the up-regulated expression of Ki67.

**Conclusions:**

Our study established an ethanol-injured corneal stroma model in both vitro and vivo. The present study confirmed that Tβ4 play a protective effect on the reconstruction process of ethanol-injured corneal stroma.

## Introduction

Ethanol is not only commonly used in clinic, but also in daily life. Similarly, most of ophthalmology surgeries would apply ethanol to sterilize the corneal surface and periorbital particularly thin skin. In addition, some types of refractive surgery, such as laser-assisted subepithelial keratectomy (LASEK), photorefractive keratectomy (PRK) and removing epithelial flap type cross linking, use 20% ethanol to loosen the connection among cornea epithelial cells and then remove the epithelium. Therefore, in this process, ethanol would speed into corneal stromal directly. Studies confirmed that ethanol could injury cells by inhibit cell differentiation and induce cell apoptosis [1, 2]. However, the corneal stroma plays an important role of maintaining corneal shape and curvature, and the corneal transparency, thus in the process of cornea stroma wound healing, the repair of corneal stroma is vital.

Originally, the thymosin proteins were discovered from fractionations of calf thymus tissue. Under natural conditions, they are short, highly charged, intrinsically unstructured proteins [3]. Though further research, thymosin family were found not only in thymus but also in other tissue, such as macrophages, tumor cells and hemocytes except erythrocyte [4, 5]. In the thymosin family, the Tβ4 is the most widely distributed among thymosin family in most of mammals. Previous studies revealed that the main function of thymosin β is inhibiting polymerization of G-actin to filaments and affects the dynamics of the cytoskeleton rearrangement through binding actin protein [6]. With the deepening of research, the Tβ4 was proved to promote cell migration, angiogenesis, differentiation of stem cells, wound healing and anti-inflammatory and anti-apoptosis [7]. It is generally known that many ocular surface diseases are associated with defect of corneal epithelium and stroma. Particularly, the condition of the corneal stroma after treatments affects the patients’ vision directly. Therefore, the one of keys to treatment these diseases are promoting the process of corneal tissue repairing. Many studies confirm that Tβ4 could promote corneal epithelial cell migration and wound healing [8–10]. Previous studies reported that Tβ4 exert its anti-inflammatory through inhibiting NFκB activity and cellular function on human corneal epithelial cell [11]. However, the research on the effect of Tβ4 on corneal stroma is comparatively limited. Corneal stromal layer is the main part of cornea. Its thickness accounts for 90% of the total thickness of cornea. It is also the main part to maintain corneal morphology, mechanical properties and corneal transparency. Significantly, corneal stroma cannot regenerate after injury instead of being substituted by scar tissue. It leads to corneal opacities that impair vision. Therefore, in the present research, in order to build a corneal stroma injury model in vitro, we applied ethanol to induce the human corneal stromal keratocyte injury. Animal model was built on BALB/c mice in vivo, and then, Tβ4’s functions were discussed in this injury model.

## Materials and methods

### Cell culture

Human corneal stromal keratocyte (HCK) were purchased from ScienCell Research Laboratories (California, US). The HCKs were recovered and cultured in Dulbecco’s Modified Eagle’s Medium (DMEM)/F12 (glutaMAX; Invitrogen/Gibco, Carlsbad, California, USA) supplementing with 10% fetal bovine serum (FBS) (Invitrogen-Gibco, Grand Island, New York, USA) and 1% mixture of penicillin and streptomycin (Invitrogen-Gibco, Grand Island, New York, USA). Every 2 days, the medium was replaced to freshly until the HCKs were cultured to reach 80 to 90% confluence. Then the cells could be passaged and transferred to medium with ethanol or Tβ4 (Invitrogen-Gibco, Grand Island, New York, USA) for further researches.

### Cell metabolic activity evaluation

Cell metabolic activity were evaluated using the Cell Counting Kit-8 (CCK-8) (Dojindo Laboratories, Japan). 1 × 10^4^ HCKs were planted onto 96-well polystyrene plates. When cells reached 70 to 80% confluence, to induce quiescence, the cells were cultured with FBS-free medium (FFM) for 12 h. Then, we performed a dose-response experiment was used to determine the ethanol concentration used in the present research, in which diluted ethanol according to a serially volume fraction (0, 5, 10, 15, 20 and 25%) was used to injury HCKs. Ethanol was diluted with phosphate buffered saline (PBS). The action time of ethanol was only 30 s and then ethanol was removed instead of culturing in FBS-free medium. The 0% group was set as control group and beyond that, a blank control (BC) was set and defined as culture medium combined with CCK-8 reagent without cells. After 48 h, the cells were incubated with the CCK-8 reagent in the dark for 2 h at 37 °C. Following the incubation, the optical density (OD) of the solution in each well was measured at 450 nm by a microplate reader (Thermo Scientific, USA). It has to be repeated at least three times. The cell metabolic activity (CM) of HCKs with different treatment were calculated using the following formula: CM = (OD - ODBC) / (ODcontrol - ODBC) × 100%.

After determining the ethanol concentration used in the present research, the HCKs were treated and grouped as follow way. ① the cells were cultured with the FFM for 48 h (control group); ② the cells were cultured in the FFM containing 1 μg/ml Tβ4 for 48 h (Tβ4 group, T); ③ the cells were treated with ethanol for 30 s and then cultured in FFM for 48 h (Ethanol group, E); ④ the cells were treated with ethanol for 30 s and then cultured in FFM containing 1 μg/ml Tβ4 for 48 h (Ethanol + Tβ4 group, E + T). Also set a blank control (BC). Then, CCK-8 assay was performed under the same procedure as mentioned above.

### Intracellular ROS measurement

The intracellular ROS levels were measured in HCKs using the redox-sensitive fluorescent dye 2′,7′-dichlorofluores-cein diacetate (DCFH-DA; Sigma-Aldrich, USA) and microscope. Briefly, HCKs were seeded onto the 24-well plate with prepared culture inserts, and then the cells in 1 × 10^5^/well of inserts at 80% ~ 90% confluence was fed FFM for 12 h. The cells were treated as the way follow, ① the cells were cultured with the FFM for 2 h (control group); ② the cells were cultured in the FFM containing 1 μg/ml Tβ4 for 2 h (Tβ4 group, T); ③ the cells were treated with 15% ethanol for 30 s (Ethanol group, E); ④ the cells were pre-treated in FFM containing 2 μg/ml Tβ4 for 2 h and then cultured with 15% ethanol for 30 s (Tβ4 + Ethanol group, T + E). Then, after 15 min, cells were treated with 10 μM DCFH-DA at 37 °C for 30 min in the dark and washed third times with PBS. Every three fields on each well were chosen to take a photo by microscope for fluorescence value (FV) measurement, Further processing of data by following formula: IRL = FV/FV_contrl_. Experiments were repeated at least three times.

### TUNEL staining assay

Terminal deoxynucleotidyl transferase dUTP nick end labeling (TUNEL) staining assay was used to detect apoptosis. The 1 × 10^5^ HCKs were seeded into the 24-well plate, the further treatments were as same as the methods and the adopted concentration applied in the CCK-8 assay. The procedures were performed in accordance with the manufacturer’s instructions strictly. In brief, the broken nuclei of the apoptosis cells were counterstained with 4′,6-diamidino-2-phenylindol. Positive cells, also means apoptotic cells were defined as the cells’ nuclear area was labeled by green. Experiments were repeated at least three times.

### ELISA assay

The levels of caspase-3 in the HCKs were detected by caspase-3 ELISA kit (R&D Systems, USA). The HCKs were cultured as the way described in the CCK-8 assay. The extract of HCKs samples and the further procedure were performed according to use instruction strictly. Experiments were repeated at least three times.

### Wound healing assays

The rate of wound closure in vitro was determined using 2-well culture insert (Ibidi, Germany) and Cytation™ 5 Cell Imaging Multi-Mode Reader (Bio Tek, USA). The culture insert is a kind of biocompatible silicone insert with a 500 μm defined cell-free gap. The HCKs were seeded into the wells of the culture insert which were placed in the center of wells of 24-well plate preparedly. Under starvation, cells were treatment as the way in the CCK-8 assay, the adopted volume fraction of ethanol was 15% (30s) and the concentration of Tβ4 was 1 μg/ml, then the inserts were moved carefully. The plate was put into the Cytation™ 5 Cell Imaging Multi-Mode Reader immediately after above works. And the program took a photo for each well every 2 h during cell migration to close the gap. The data was further calculated by the following formula: Growth rate (GR) = Cell Area/total area× 100%.

### Animal model of corneal injury and treatment

Twenty-four eyes of twelve BALB/c mice were studied in these experiments. Animal were aged 6-8 weeks. All mice in the experimental group were anaesthetized with a mixture of ketamine (50 mg/kg) and xylazine (10 mg/kg) by intraperitoneal injection. 98% ethanol was applied to the central cornea (a diameter of 2.5 mm) for 15 s, followed by rinsing with 1 ml of phosphate-buffered saline (PBS). Then, the epithelium over the injury area was mechanically scraped using a surgical blade. A corneal wound which exposes cornea stroma was generated in the right eye of each mouse. Then, in order to fruiting cornea stroma injury model, 20% ethanol was used to cover a diameter of 2 mm stroma area for 30s.

Twenty-four eyes of twelve mice were randomly divided into four groups. The control group (*n* = 6 eyes) and Tβ4 group (T, *n* = 6 eyes), of which the cornea was normal, were administered with placebo and Tβ4 (5μg/5ul) four times per day respectively. Immediately following injury, the group was applied as follows: mice received Tβ4 (Ethanol + Tβ4 group, E + T, *n* = 6 eyes) or placebo (Ethanol group, E, *n* = 6 eyes).

Every mouse’s right eye was injured by ethanol and left eye was normal, treatments were performed for 3 days. During this period, the area of epithelial defect was stained with a drop of 0.05% fluorescent strip and used a slit-lamp biomicroscope under a cobalt blue light for capturing images. For the injured area of the cornea was measured using the software Image Pro-Plus V.6.0 and calculated as a percentage of the residual epithelial defect.

### H&E staining and immunofluorescent staining

Seventy-two hours after ocular damage, all mice were sacrificed by intraperitoneal injection of sevoflurane anesthesia and cervical dislocation. Mice eyeballs were removed while both H&E staining and immunofluorescent staining was performed. The eyeballs were fixed in formalin, then prepared in paraffin-embedded blocks for sectioning at a thickness of 4 μm for routine histological processing. Tissue sections were stained with hematoxylin and eosin (H&E). For immunofluorescence, mice eyeballs were fixed in 4% paraformaldehyde (PFA) for 15 mins, then embedded and snap frozen in liquid nitrogen. Sections of 4.5 μm were mounted onto cryostate (Leica CM 1850; Leica Microsystems GmbH, Wetzlar, Germany) and incubated with rabbit monoclonal antibody against ki67 (1:500, Catalogue # MAB7617, R&D Systems, MN, USA) at 4 °C overnight, followed by incubation with DyLight 594-conjugated goat anti-rabbit IgG (1:200, Thermo Fisher Scientific, Inc.) at room temperature for 1 h. Nuclei were stained with 4,6-diamidino-2-phenylindole (DAPI, 1:1000, Beijing Solarbio Science & Technology Co., Ltd., cat. no. S2110). And the sections were observed by Cytation™ 5 Cell Imaging Multi-Mode Reader (Bio Tek, USA). The number of positive cells of corneal stroma in the visual field of each group was counted.

### Real-time quantitative PCR

1 × 10^6^ HCKs were planted onto 6-well plates and treated by the way described in the CCK-8 assay. Total RNAs of HCKs and cornea tissue of BALB/c mice were extracted using Qiagen RNeasy Mini Kit (Qiagen, Hilden, Germany) according to the manufacturer’s instructions and then reverse-transcribed into cDNA. For the cornea of animals, two pieces of cornea were detected each time in each group randomly and repeated three times. The real time quantitative PCR amplification was done for 45 cycles in the following thermal cycle using SYBR Green (Invitrogen): 95 °C for 15 s, 60 °C for 1 min, and 95 °C for 15 s. The primer sequences were as follows: hGAPDH (forward, 5′-CCACTCCTCCACCTTTGACG-3′, reverse, 5′-TAGCCAAATTCGTTGTCATACCAGG-3′); hCaspase-3 (forward, 5′-AGCGAATCAATGGACTCTGGA− 3′, reverse, 5′-GGTTTGCTGCATCGACATCT− 3′); hCatalase (forward, 5′-CTCCGGAACAACAGCCTTCT-3′, reverse, 5′- GATGAGCGGGTTACACGGAT-3′); hBcl-2 (forward, 5′-TGGAGGAGCTCTTCAGGGA-3′, reverse, 5′- CAGCCTCCGTTATCCTGGAT-3′); hCuZnSOD (forward, 5′- GCAGATGACTTGGGCAAAGG-3′, reverse, 5′- GGGGCCTCAGACTACATCCA-3′). mGAPDH (forward, 5′- ATACGGCTACAGCAACAGGG-3′, reverse, 5′- GCCTCTCTTGCTCAGTGTCC-3′); mCaspase-3 (forward, 5′- TGTGTCCATGCTCACGAAAG-3′, reverse, 5′- AAAGACCAGGAGGACCGTCA-3′); mBcl-2 (forward, 5′-AGTACCTGAACCGGCATCTG-3′, reverse, 5′-TATGCACCCAGAGTGATGCAG-3′).

### Statistical methods

Data were summarized using mean ± SD values and analyzed using IBM SPSS Statistics 24 statistical software (SPSS, Inc., USA). An equality of variances was determined by Levene’s test. A Student t-test was used to assess the differences between the two means. The assessment of multiple means was performed by one-way or two-way ANOVA followed by the Bonferroni’s post hoc test. A *p* value of < 0.05 was considered significant.

## Results

### Effect of ethanol and Tβ4 on the cell metabolic activity of HCKs

To investigate the effect of various concentrations of ethanol and Tβ4 on the cell metabolic activity of HCKs, we performed a dose–response experiment, in which serially diluted ethanol (0, 5, 10, 15, 20 and 25%) was added to injury HCKs, and beyond that, HCKs treated with 15% ethanol was applied to culture in medium supplementing with 1 μg/ml Tβ4.

**Fig. 1:**
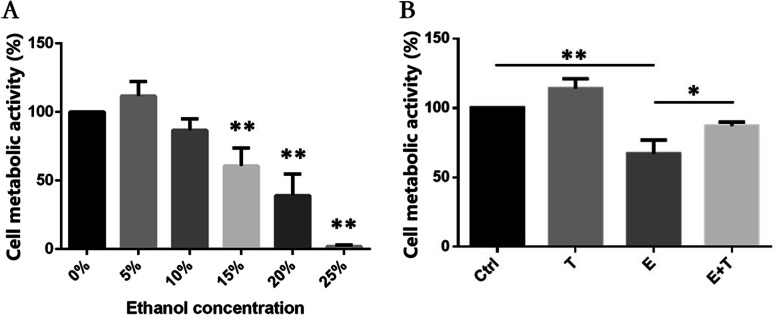
The results of CCK-8. **A** showed variation cell metabolic activity of HCKs in different groups. **B** showed the cell metabolic activity of HCKs with ethanol and/or Tβ4 treatment. *p < 0.05, **p < 0.01

The results of CCK-8 showed that low concentration of ethanol (5%) resulted in higher cell metabolic activity (111.7 ± 6.06%) than the control group (100 ± 0.00%). Besides, cell metabolic activity was on the decline with the increased concentration of ethanol (86.7 ± 6.80%, 60.7 ± 10.62%, 39.0 ± 12.96%, 2 ± 0.82%) (Fig. 1A, *F* = 50.95, *p* < 0.01). Ethanol of certain concentrations could present viability inhibition effects on HCKs. 15% ethanol treated HCK, of which the value of CCK-8 was 0.61-fold than control (*p* < 0.01), was chosen to applied for a series of further experiments. The results showed that 1 μg/ml Tβ4 could enhance viability of cells which were treated with 15% ethanol (Ctrl 100.0 ± 0.00%, T 113.7 ± 4.99%, E 60.7 ± 10.62, E + T 87.7 ± 4.19%), (Fig. 1B; *p* < 0.05).

### Effect of ethanol and Tβ4 on ROS generation of HCKs

According to the results of CCK-8 assay, it confirmed that 15% ~ 25% ethanol cause HCKs injury. To explore which type of injury does ethanol induce, the 15% ethanol and/or 1 μg/ml Tβ4 treated HCKs were adopted for intracellular ROS and apoptotic cell measurement.

**Fig. 2:**
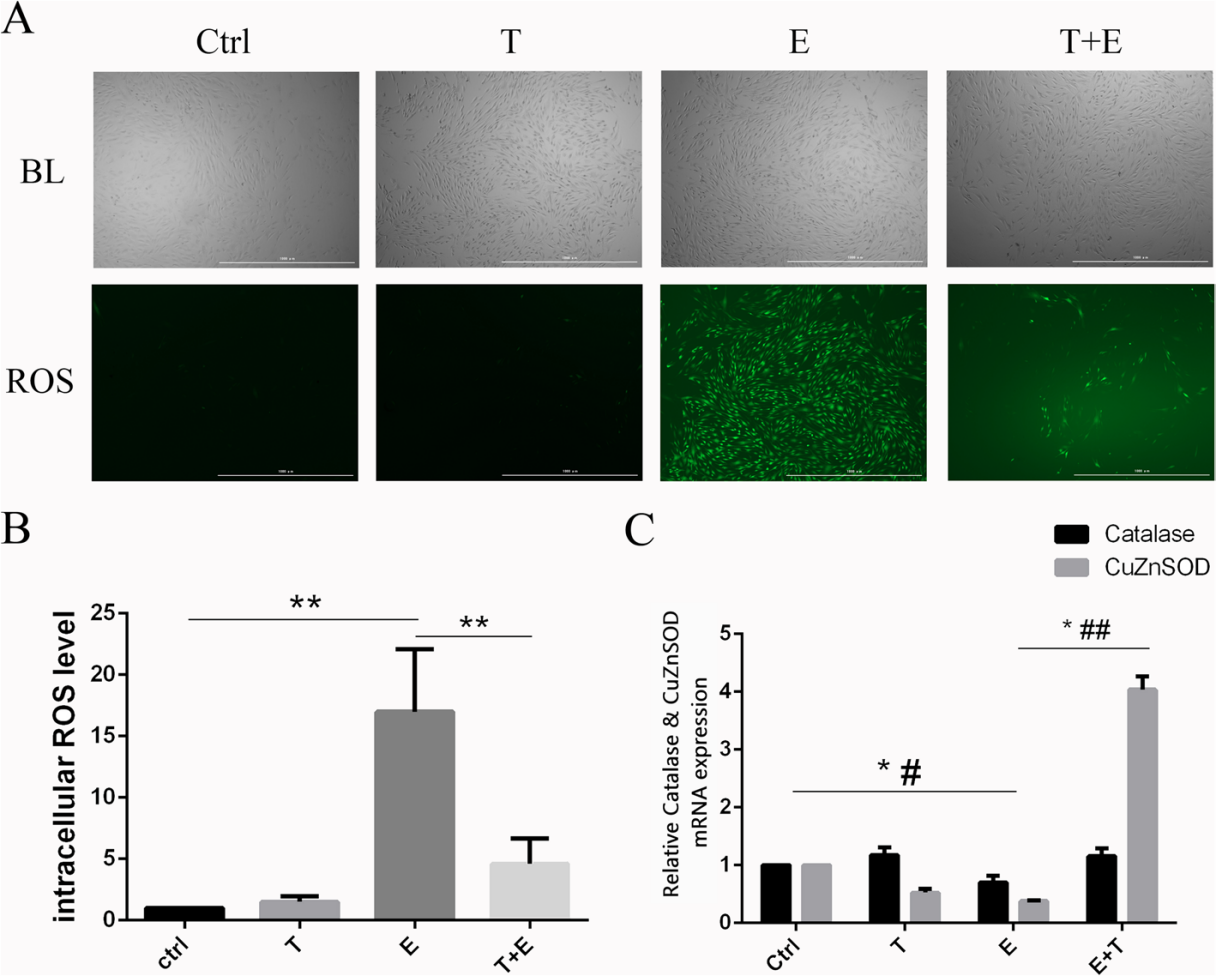
The results of ROS. **A** showed the intracellular ROS of four groups, Scale bar: 1000 μm. **B** showed the fluorescence values (FV) of HCKs, IRL = FV/FV_contrl_. **C** showed the expression of Catalase and CuZnSOD of HCKs. * *p* < 0.05(Catalase), # *p* < 0.05(CuZnSOD), ## *p* < 0.01(CuZnSOD) BL: bright light field

The mean fluorescence intensity observed in ROS measurement assay is a true measure of the intracellular ROS level (IRL) of HCKs. The results showed that the IRL of cells in E group (16.98 ± 4.17) was significantly higher than control group (1.00 ± 0.00) (*p* < 0.01). Pre-treatment of ethanol injury HCKs with Tβ4 (T + E group) (4.61 ± 1.68) significantly declined the IRL compared with it of E group (*p* < 0.01). These results suggested that ethanol could induce generation of ROS on HCKs and cause oxidative stress injuries and Tβ4 could inhibit it effectively (Fig. 2A and B).

### Effect of ethanol and Tβ4 on the expression of oxidative stress related gene on HCKs

In order to study the correlated mechanism of ethanol and Tβ4 on HCKs’ oxidative stress damage, we detected the relative quantitation of Catalase, CuZnSOD. The results of qPCR revealed that the relative quantitation of Catalase and CuZnSOD of HCKs of E group was down-regulated (0.70 ± 0.10, 0.38 ± 0.01) (*P <* 0.05, *P <* 0.01). Besides that, Tβ4 up-regulated Catalase and CuZnSOD genes on the ethanol injury HCKs (E + T group) (1.16 ± 0.11, 4.04 ± 0.19) (*P <* 0.05, *P <* 0.01) (Fig. 2C).

### Effect of ethanol and Tβ4 on cell apoptosis

**Fig. 3:**
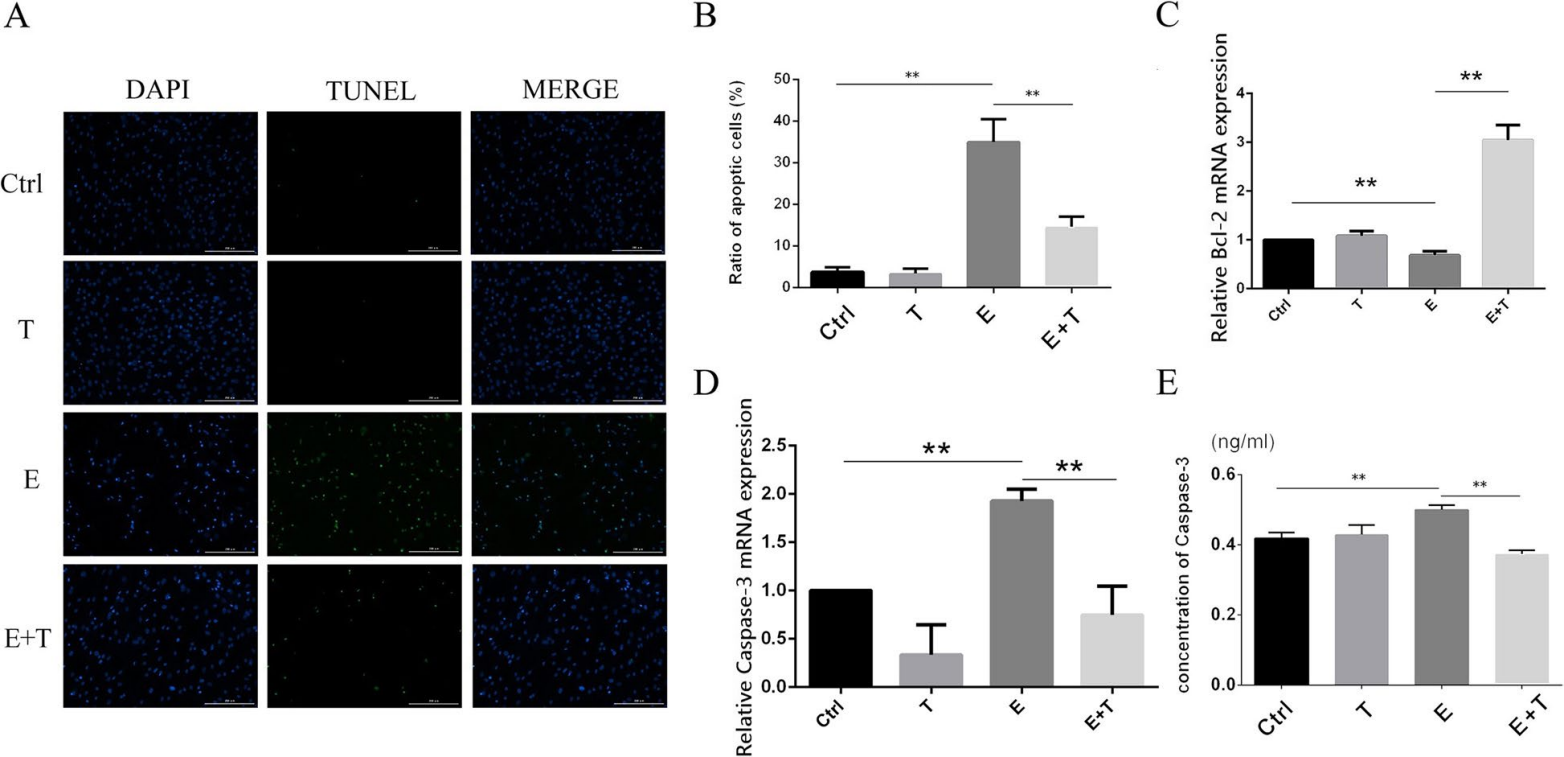
The results of cell apoptosis. **A** showed the cell apoptosis of four groups by TUNEL staining, Scale bar: 200 μm. **B** showed the result of positive cell (green) count. **C** showed the expression of Bcl-2 of HCKs detected by qPCR. **D** showed the expression of caspase-3 of HCKs detected by qPCR. **E** showed the protein level of caspase-3 of HCKs by ELISA. **p < 0.01

Besides IRL measurements, we also performed the cell apoptosis measurement assay by TUNEL assay. The rate of apoptotic cell (the cells of which nuclear are green) were used as indicators of cell apoptosis. The results showed that treatment of HCKs with 15% ethanol for 30 s resulted in increased numbers of apoptotic cells (E group, 0.35 ± 0.03) than control group (0.04 ± 0.01). The apoptotic cell rate of E group was 8.75-fold of it of control group, the results had significant difference between the two groups (*p* < 0.01). Besides that, the results also revealed that Tβ4 can effectively inhibit the apoptosis of ethanol injury HCKs. The apoptotic cell rate of E + T group (0.15 ± 0.01) decreased significantly compared with it of E group, the difference was significant (*p* < 0.01) (Fig. 3A and B). The protective effects of Tβ4 produced mainly in the inhibition of ethanol treated HCKs apoptosis.

### Effect of ethanol and Tβ4 on the expression of cell apoptosis related gene on HCKs

To further characterize the effects of ethanol and Tβ4 on HCKs apoptosis, we assessed the nucleic acid expression of vital apoptotic related gene, Caspase-3 and Bcl-2, in HCKs of different group were measured by qPCR. In addition, the protein levels of Caspase-3 were measured by ELISA.

The results of qPCR showed that the relative quantitation of Caspase-3 in HCKs cells of E group (1.93 ± 0.10) was significantly increased compared to it of control group (1.00 ± 0.00) (*p* < 0.01), but the relative quantitation of Caspase-3 of E + T group (0.75 ± 0.24) was less than it of E group (Fig. 3D) (*p* < 0.01). The trends of Bcl-2 (ctrl 1.00 ± 0.00, T 1.08 ± 0.08, E 0.68 ± 0.08, E + T 3.04 ± 0.25) (*p* < 0.01) on the above three groups are opposite to it of Caspase-3 (Fig. 3C). Beyond that, the results of ELISA for Caspase-3 suggested that the trends of Caspase-3 on protein level (Ctrl 0.42 ± 0.01 ng/ml, T 0.43 ± 0.02 ng/ml, E 0.50 ± 0.01 ng/ml, E + T 0.37 ± 0.01 ng/ml) (*p* < 0.01) in the different groups matched to it on nucleic acid level (Fig. 3E).

### Effect of ethanol and Tβ4 on HCKs wound healing in vitro

**Fig. 4:**
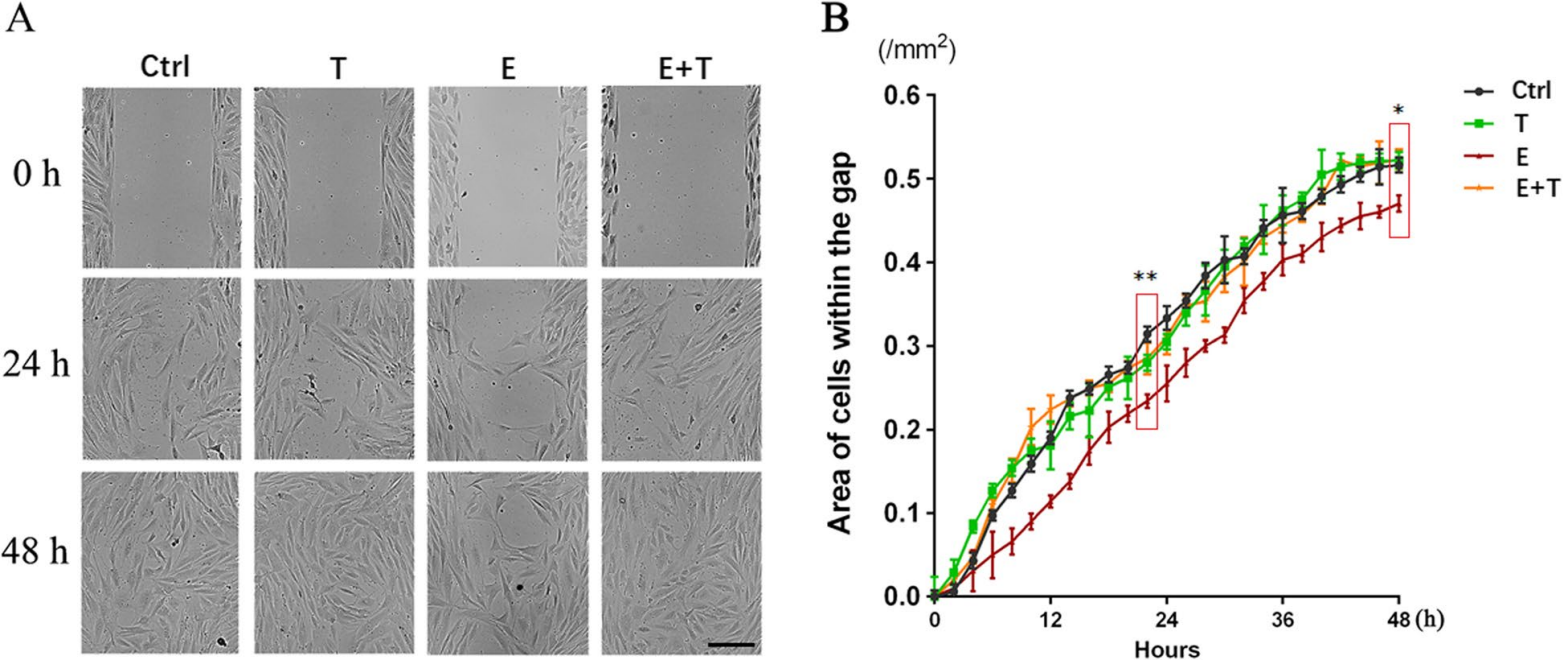
The results of wound healing in vitro. **A** showed the condition of gap at three time points (0 h, 24 h, 48 h), (10×). Scar bar: 200 μm. **B** showed the area of cells within the gap of four groups

Based on these results, we tested whether Tβ4 play a role on HCKs’ proliferation and migration by in vitro experiments. At the beginning time point, The GR of four groups were not statistically significant (Ctrl 19.37 ± 4.72%, T 15.69 ± 3.65%, E 15.84 ± 3.44%, E + T 15.65 ± 3.51%) (*p* > 0.05). Gap closure in the control group began 4 h after the scratch. After 24 h, the results revealed that migration ability of the cells of E group (54.24 ± 12.61%) was lower than the control group (71.33 ± 7.94%) (*p* < 0.01). At the last time point (after 48 h), HCKs layer of control group (92.95 ± 8.15%), T group (80.12 ± 12.03), and E + T group (88.70 ± 1.83%) had almost 100% closure. In contrast, the cell layer of E group just growth to (69.65 ± 8.24%) closure (Fig. 4A and B) (*p* < 0.05).

### Tβ4 enhances corneal wound healing and corneal stroma restoration

**Fig. 5:**
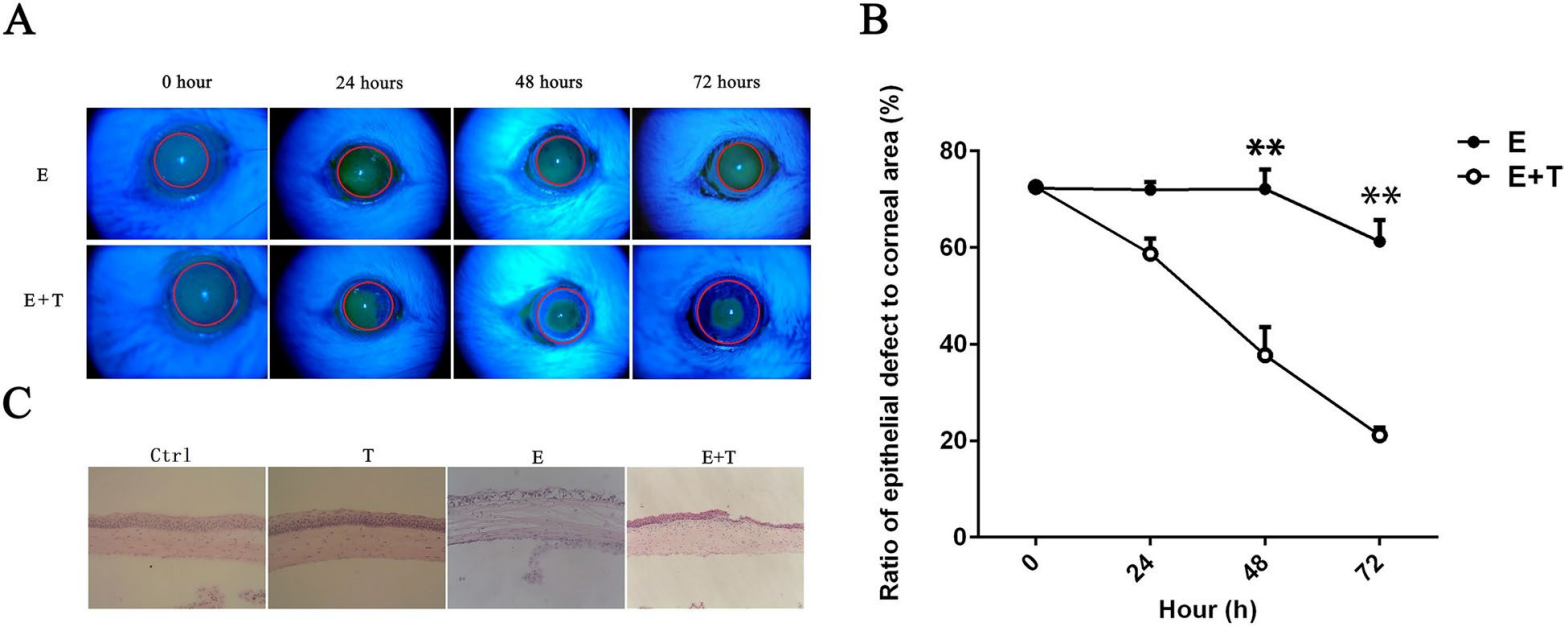
The results of wound healing in vivo. **A, B** the fluorescence staining showing epithelial defect of cornea of four groups. The red circle means the baseline of corneal epithelial defects area. The green part means the epithelial defect area. **C** showed the HE staining of cornea of four groups (10×)

To further certify that Tβ4 enhances both corneal epithelial and stroma wound healing, we established a well-characterized sterile injury murine model. In the E + T group, after ethanol injury of the stroma, Tβ4 was applied topically. Compare with the E group, sterile PBS was applied after the damage. We used corneal fluorescein staining to monitor the defect of epithelial, which could present the situation of the wound healing. At the beginning time point, ratio of epithelial defect to corneal area between E (72.85 ± 1.53%) and E + T (72.60 ± 1.46%) group without significant different (*p* > 0.05). A significant accelerate of epithelial regeneration was observed in eyes treated with Tβ4 comparing with the E group on day 2 (E 71.81 ± 1.08%, E + T 37.98 ± 1.90%) and day 3 (E 61.17 ± 2.86%, E + T 21.00 ± 1.90%) (*p* < 0.01) (Fig. 5A and B). According to the histological evaluation, severe cornea edema was found in both corneal epithelial and stroma of the central cornea in E group, which also showed moderate inflammatory cells infiltration. Tβ4 significantly ameliorated corneal edema of both corneal epithelial and stroma, while increased inflammatory cells infiltrating the anterior stroma of the central cornea in the E + T group. In addition, the epithelial basal cells’ arrangement of T group was more regular and the number of them were more than did the control group (Fig. 5C).

### Tβ4 enhance cell proliferation on both epithelial and stroma

**Fig. 6:**
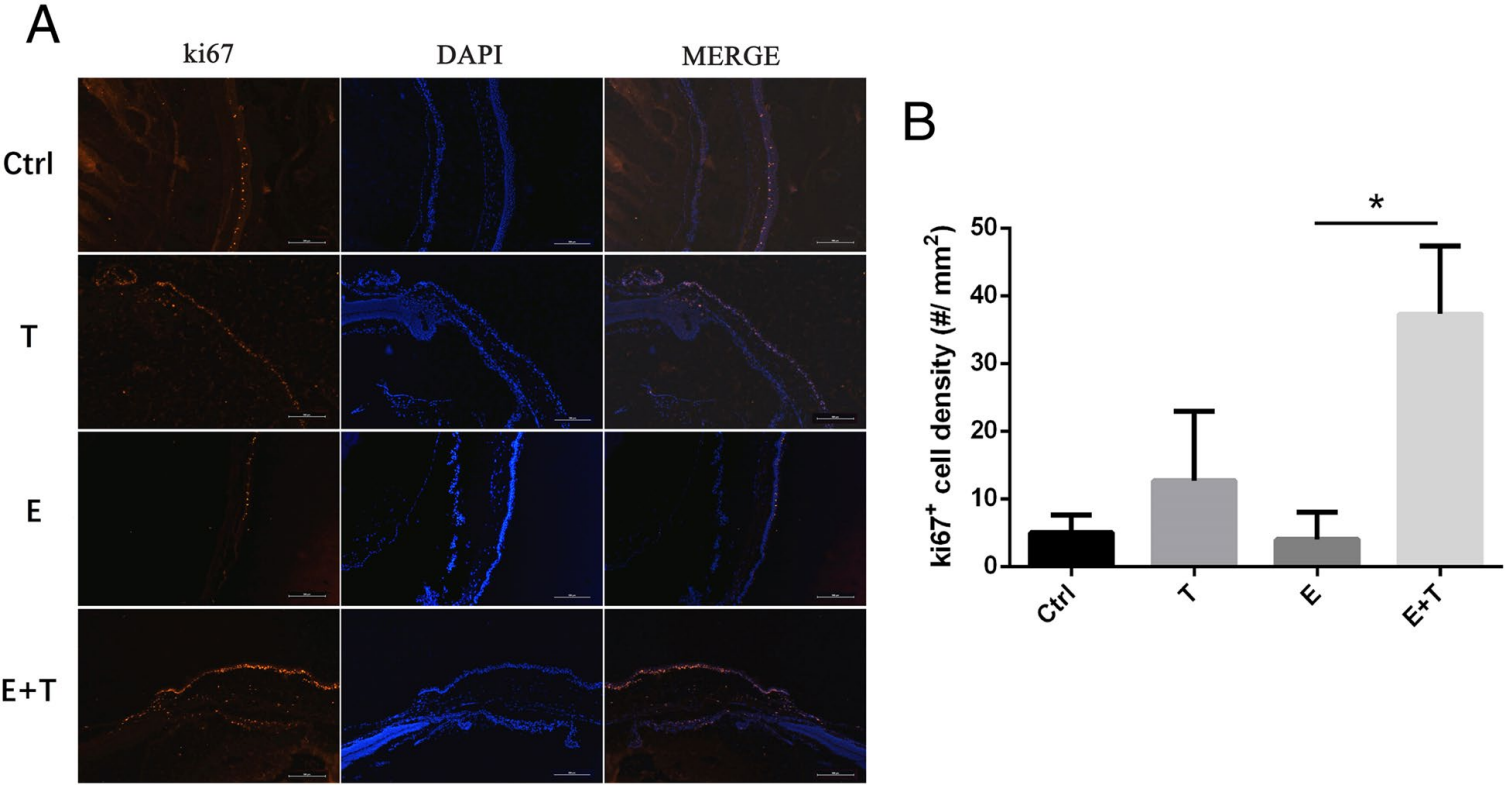
**A** The results of ki67 on corneal tissue. Red showed the expression of Ki67, blue = DAPI. Scale bar: 500 μm. **B** Positive cell count of corneal stroma of four groups. *p < 0.05

Our in vitro studies demonstrated that Tβ4 plays a vitally important role in corneal stroma cell proliferation, thereore we investigated whether Tβ4 could also accelerate the regeneration of corneal stroma cell in vivo. To determine whether the cell regeneration in the corneal stroma could increase due to Tβ4 treatment, the expression of ki67, markers of cellular proliferation were examined. ki67-expressing cells were found in the basal of corneal epithelial and corneal stroma, which both increased obviously in the E + T group comparing with the E group (Fig. 6A&B). However, in the E group, the regeneration of corneal cells showed the least. Tβ4 treatment enhanced cell regeneration in T groups than control group (Fig. 6A&B).

### Expression of cell apoptosis related gene on animal model

**Fig. 7:**
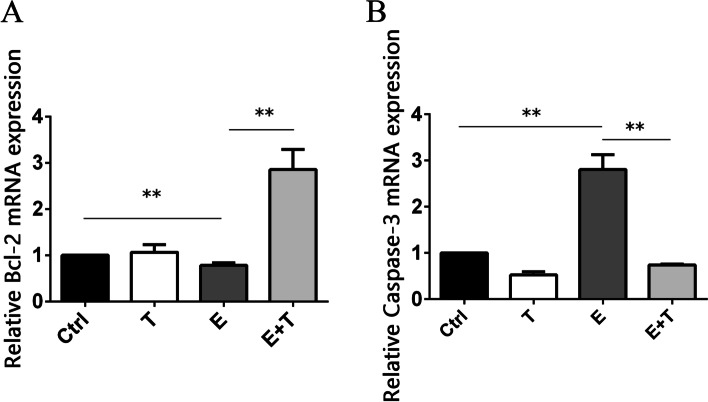
The results of cytokine correlating cell apoptosis on animal model. **A** showed the expression of Bcl-2 of BALB/c mice cornea tissue detected by qPCR. **B** showed the expression of caspase-3 of BALB/c mice cornea tissue detected by qPCR. **p < 0.01

In order to validate the effect of ethanol and Tβ4 on apoptosis in vivo, we also detected the relative quantity of Bcl-2 and Caspase-3 on corneal tissue of BALB/c mice. The results showed that, on the animal model, the trends of the relative quantity of the above two mRNA gene of the four groups were similar to that of cell levels. Compared to the control group (1.00 ± 0.00), the relative quantity of Bcl-2 of E group (0.78 ± 0.04) was declined (*p* < 0.01). And it of E + T group (2.86 ± 0.35) was higher than other three group (*p* < 0.01, Fig. 7A). The trends of Cspase-3 of four groups were also similar to the cell levels. The relative quantity of Caspase-3 value of E group (2.81 ± 0.26) was increased than it of control group (1.00 ± 0.00) (*p* < 0.01). And it of E + T group (0.74 ± 0.02) was about 0.26-fold of it of E group (*p* < 0.01, Fig. 7B). The above results further strengthened the anti-apoptotic function of Tβ4.

## Discussion

The present study was aimed to explore ethanol injury on HCKs and its mechanism. More significantly, we investigated whether Tβ4 treatment will prevent the HCKs from the ethanol injury. The actions and mechanism of Tβ4 in the repairing HCKs and corneal stroma injury were also researched. The results showed that the ethanol can result in oxidative stress and cell apoptosis of HCKs. Tβ4 can effectively alleviate the above injuries in in vitro model and promote the corneal wound healing in in vivo model.

Corneal stromal wound healing is a complex event includes corneal keratocytes apoptosis, and trans-differentiation into fibroblasts. Therefore, although corneal keratocytes take fibroblast phenotype in the presence of serum in our research, it also could be considered as one of phenotype of corneal keratocytes. It is consistent with the repair process of corneal stromal injury.

Study of Y Su et al. demonstrated a strong association between Tβ4 and intracellularly biochemical reactions like oxidative stress, and cell apoptosis on human corneal epithelial cells in vitro [12]. The corneal stromal wound healing is a complex process, keratocytes are key mediators of it. Therefore, based on the previous study, in the present study, we selected HCKs as the research object and detected the cell metabolic activity firstly.

The results demonstrated that ethanol treatment reduced the cell metabolic activity of HCKs, and Tβ4 increased the cell metabolic activity of HCKs following ethanol injury. Therefore, the results of the present study suggested that ethanol could injury the HCKs and Tβ4 could protect cells from the damage. Consequently, we further explored the mechanism of action of ethanol and Tβ4 in the process.

Whether clinical or experimental researches, there are less studies focusing on ethanol-induced ROS generation on corneal cells. However, on liver cells, the generation of ROS induced by ethanol has been studied extensively [13–15]. We detected the intracellular ROS levels of four groups HCKs. The results stated that the IRL of HCKs treated by ethanol was increased, but it of Tβ4 treatment HCKs following ethanol was decreased. Beyond that, the qPCR results could explain it. The results of qPCR revealed that ethanol down-regulated the express of Catalase and CuZnSOD of HCKs, but the expression of the above two gene were increased on HCKs of T + E group. Both Catalase and CuZnSOD are antioxidant enzyme. Comprehensive above results, ethanol could induce the generation of ROS on HCKs, and Tβ4 could eliminate them by regulating the expression of relative enzymes. The results are consistent with the study of Y Su et al. [12] In addition, there are many studies suggested that many cornea diseases are relative to oxidative stress of corneal tissue, such as cornea injury (including chemical injury and ultraviolet irradiation), bullous keratopathy, and keratoconus [16–18]. Therefore, this function of Tβ4 is especially meaningful to clinical treatment of the above cornea diseases.

Recent researched indicated that intracellular oxidative stress could lead to cell apoptosis. In our present study, we found that ethanol treatment induced HCKs apoptosis. Tβ4 could inhibit cell apoptosis by regulating the expression of Bcl-2 and Caspase-3. Many studies have proven the efficacy of Tβ4 to inhibit cell apoptosis on various tissue and cells [19, 20]. There are also many studies verifying that Tβ4 have significant anti-apoptosis effect on corneal tissue [21, 22]. The results stated that Tβ4 not only alleviate the oxidative stress injury, but also inhibit cell apoptosis induced by oxidative stress.

In addition, through wound healing assay in vitro, we confirmed that Tβ4 could promote the HCKs proliferation and migration. In order further to prove the results, animal model was established to verify its efficacy. The results demonstrated that efficacy of Tβ4 to promote corneal epithelial and stroma wound healing was significant. The results also showed that Tβ4 worked through upregulated the expression of ki67. Ki67 is identified as a proliferation-associated nuclear antigen. It had been proved that ki67 express during the G1, S, and G2 phases of cycling cells. Thus, ki67 is considered as a proliferation marker [23]. The results of our present study showed that Tβ4 could upregulated the expression of ki67 on the tissue of corneal stroma following the ethanol treatment, and promote the corneal stroma repairment.

In summary, we performed in vitro and in vivo assay to verify Tβ4’s role protection cells and tissue from ethanol injury and promote the corneal stroma wound healing on the HCKs and animal models. Our present data demonstrate that Tβ4 could regulated the expression of Catalase, CuZnSOD, Bcl-2 and Caspase-3 to eliminate excessive ROS and protect membranes from oxidation and oxidative-induced cell apoptosis. Moreover, we performed both in vitro and in vivo assay to explore the function of Tβ4 to promote the corneal wound healing. Therefore, Tβ4 protects HCKs from ethanol-induced oxidative stress and cell apoptosis, and significantly promote the corneal stroma wound healing.

## Data Availability

The data supporting our findings is contained in within the manuscript and figures. The datasets used and/or analysed during the current study are available from the corresponding author on reasonable request.

## Abbreviations

Tβ4: Thymosin β4
HCKs: Human corneal keratocytes
CCK-8: Cell Counting Kit-8
DCFH-DA: 2′,7′-dichlorofluores-cein diacetate
OD: Optical density
CM: Cell metabolic activity
FV: Fluorescence value
TUNEL: Terminal deoxynucleotidyl transferase dUTP nick end labeling
GR: Growth rate
ELISA: Enzyme linked immunosorbent assay
